# Cor triatriatum with supramitral ring: “cor tetratriatum”, associated with Raghib syndrome with Eisenmenger syndrome: multimodality imaging approach in this exceedingly rare case report

**DOI:** 10.1186/s43044-021-00183-4

**Published:** 2021-07-17

**Authors:** Barun Kumar, Ashwin Kodliwadmath, Amar Nath Upadhyay, Anupam Singh, Anshuman Darbari, N. Nanda

**Affiliations:** 1 Department of Cardiology, All India Institute of Medical Sciences (AIIMS), Virbhadra road, Rishikesh, Uttarakhand 249203 India; 2 Department of Cardiology, PGIMER, Dr Ram Manohar Lohia Hospital, Delhi, India; 3 Doon Medical College, Dehradun, Uttarakhand India; 4 Department of Ophthalmology, All India Institute of Medical Sciences (AIIMS), Rishikesh, Uttarakhand India; 5 Department of CTVS, All India Institute of Medical Sciences (AIIMS), Rishikesh, Uttarakhand India; 6 Department of Endocrinology, All India Institute of Medical Sciences (AIIMS), Rishikesh, Uttarakhand India

**Keywords:** Cyanosis, Congenital heart disease, Persistent left superior vena cava, Unroofed coronary sinus, Eisenmenger syndrome, Raghib syndrome, Supramitral ring, Cor triatriatum, Cor tetratriatum, Case report

## Abstract

**Background:**

Cor triatriatum and supramitral ring are congenital anomalies which result in formation of three chambers of atria. To the best of our knowledge, simultaneous presence of both entities in the same patient resulting in the formation of four chambers of atria has not been described in the literature. Here, we report a case of simultaneous presence of cor triatriatum and supramitral ring associated with Raghib syndrome and Eisenmenger syndrome.

**Case presentation:**

We report the case of a middle-aged gentleman, who presented to us with features of atrial septal defect with Eisenmenger syndrome. Multimodality imaging confirmed the simultaneous presence of supramital ring and cor triatriatum resulting in “cor tetratriatum” along with Raghib syndrome. Presence of Eisenmenger syndrome compelled us to offer medical therapy for the patient.

**Conclusion:**

This is the first case report describing the simultaneous presence of supramitral ring and cor triatriatum resulting in a new entity—“cor tetratriatum”.

**Supplementary Information:**

The online version contains supplementary material available at 10.1186/s43044-021-00183-4.

## Background

Cor triatriatum sinister and supramitral ring are congenital anomalies which result in formation of three chambers of atria by a membrane in the left atrium (LA). The position of the membrane differentiates the two entities. In supramitral ring, the membrane is present below the left atrial appendage (LAA), while it is above the LAA in cor triatriatum [1]. To the best of our knowledge, simultaneous presence of both entities in the same patient resulting in the formation of four chambers of atria has not been described in the literature. Here, we report a case of simultaneous presence of cor triatriatum and supramitral ring associated with Raghib syndrome and Eisenmenger syndrome.

## Case presentation

**Fig. 1:**
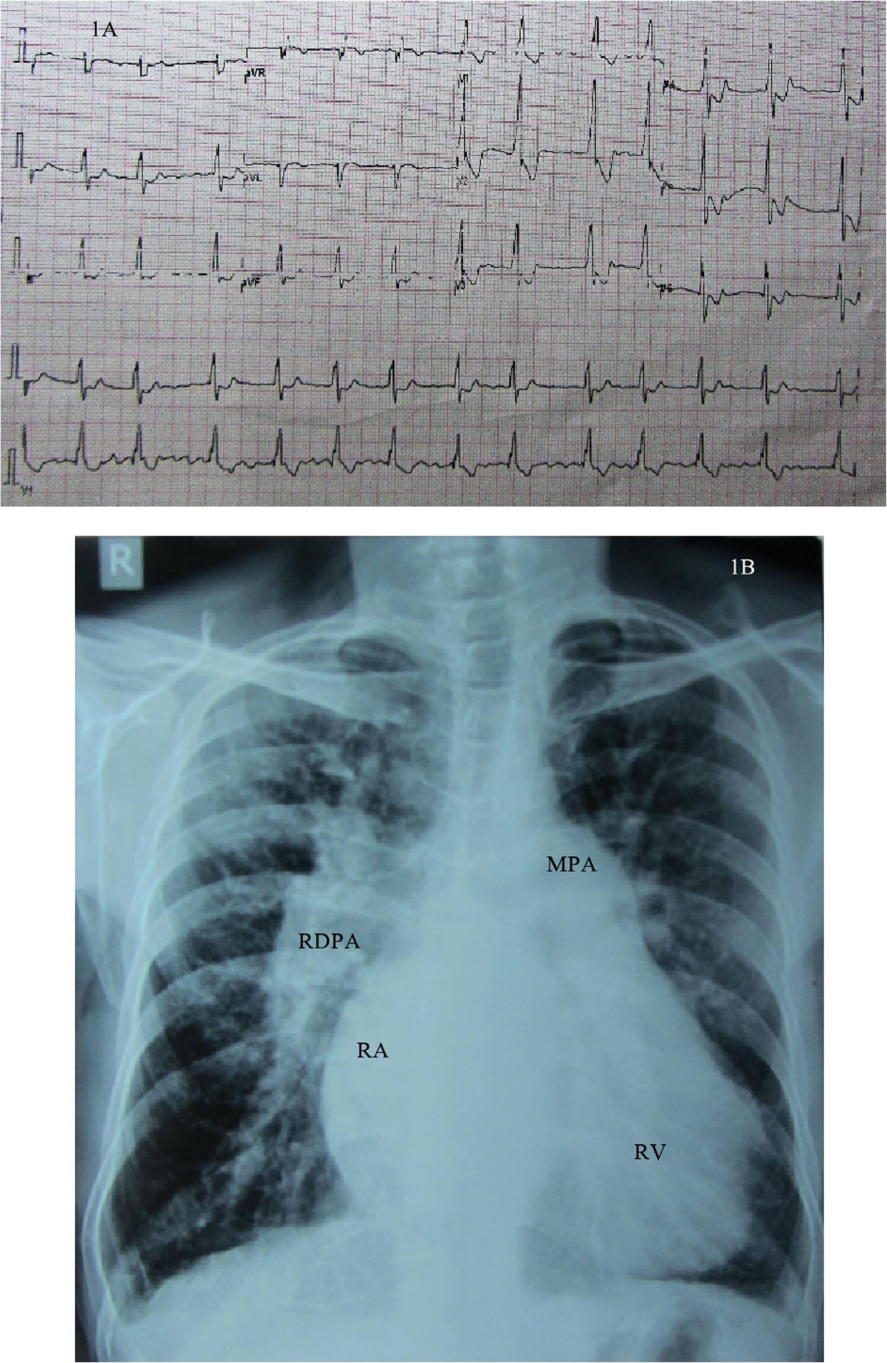
Twelve lead surface electrocardiogram showing right bundle branch block with atrial fibrillation (**A**) and chest X-ray posteroanterior view showing situs solitus, levocardia, right atrial enlargement, right ventricular apex, dilated main and right descending pulmonary arteries, and peripheral pruning of pulmonary vessels (**B**). RA, right atrium; RDPA, right descending pulmonary artery; MPA, main pulmonary artery; RV, right ventricle

**Fig. 2:**
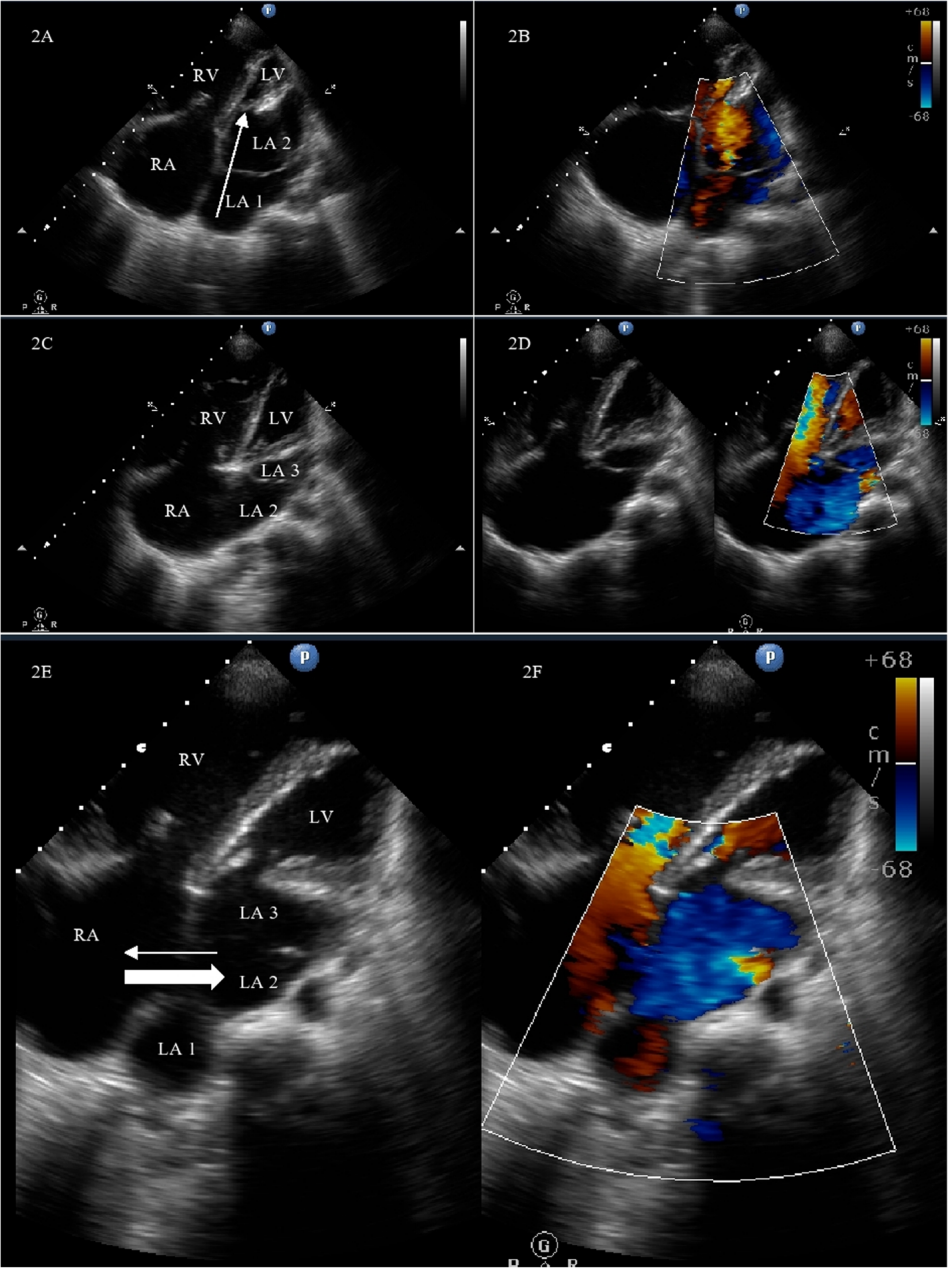
Transthoracic echocardiography in apical 4 chamber (A4C) view showing left atrium (LA) divided into 2 chambers LA1 and LA2 by a membrane (**A**) with color Doppler showing pulmonary venous return progressing from proximal left atrium to left ventricle through the membrane (arrow) (**B**). Modified A4C view opening up the 3rd LA chamber (LA3) separated from LA2 by the supramitral ring with color Doppler showing flow through the supramitral ring (**C**, **D**). Modified A4C view showing all 3 LA chambers with LA2 communicating with right atrium through the atrial septal defect (**E**, **F**). The thicker arrow shows predominant right to left shunt, and the thinner arrow shows the small left to right shunt. RA, right atrium; LA, left atrium; RV, right ventricle; LV, left ventricle

**Fig. 3:**
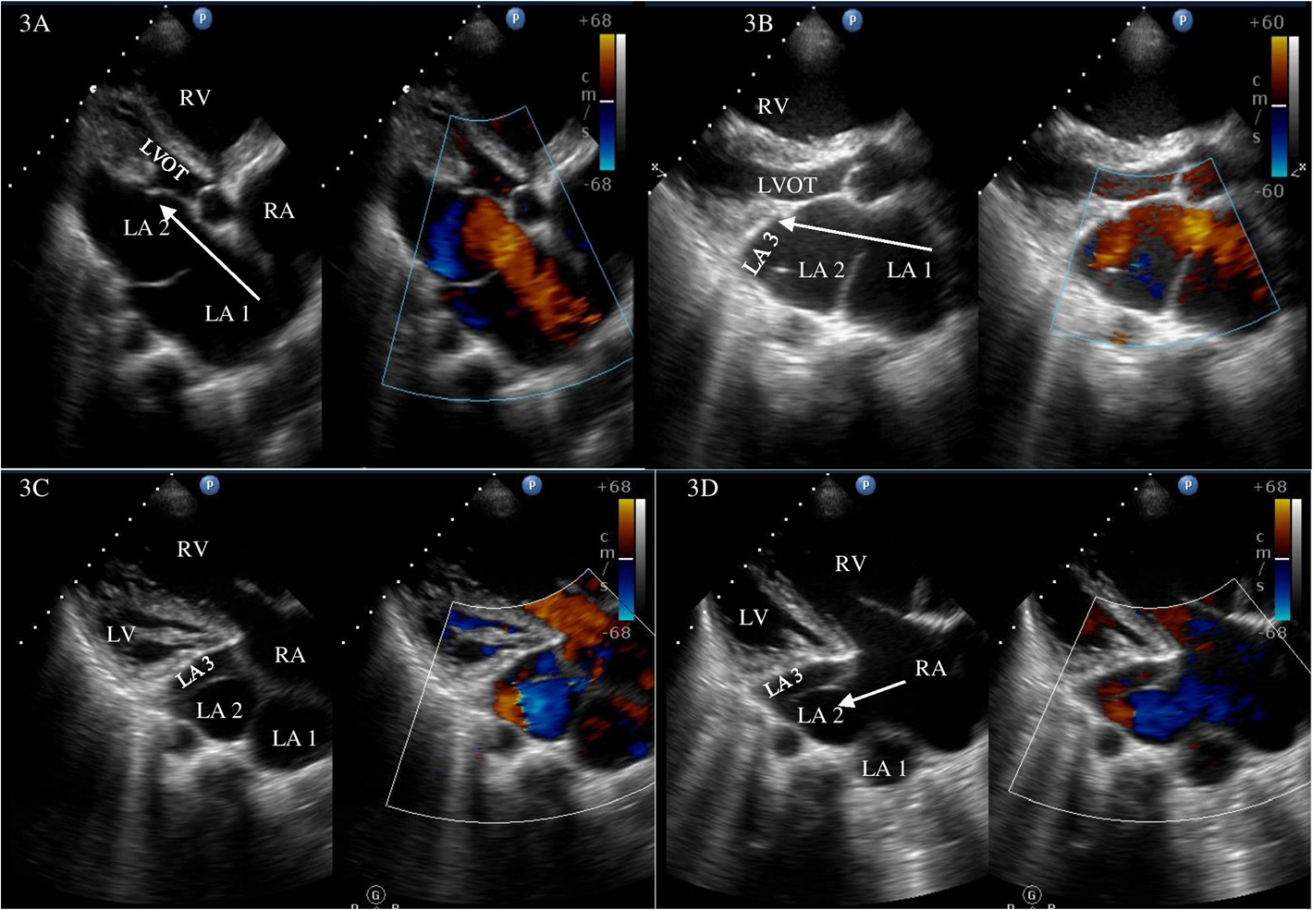
Transthoracic echocardiography in parasternal long axis (PLAX) view showing left atrium (LA) divided into 2 chambers by a non-obstructive membrane with color Doppler showing uninterrupted blood flow from LA1 to LA2 (**A**). Modified PLAX view showing 2 membranes in the LA diving it into 3 chambers with uninterrupted blood flow from LA1 to LA3 (**B**). Arrow in **A** and **B** showing the direction of flow of blood. Modified PLAX view showing all 3 chambers in LA with presence of ASD between LA2 and right atrium with predominant right to left shunt (**C**, **D**). Arrow in **D** shows the right to left shunt. RA, right atrium; LA, left atrium; RV, right ventricle; LV, left ventricle; LVOT, left ventricular outflow tract

**Fig. 4:**
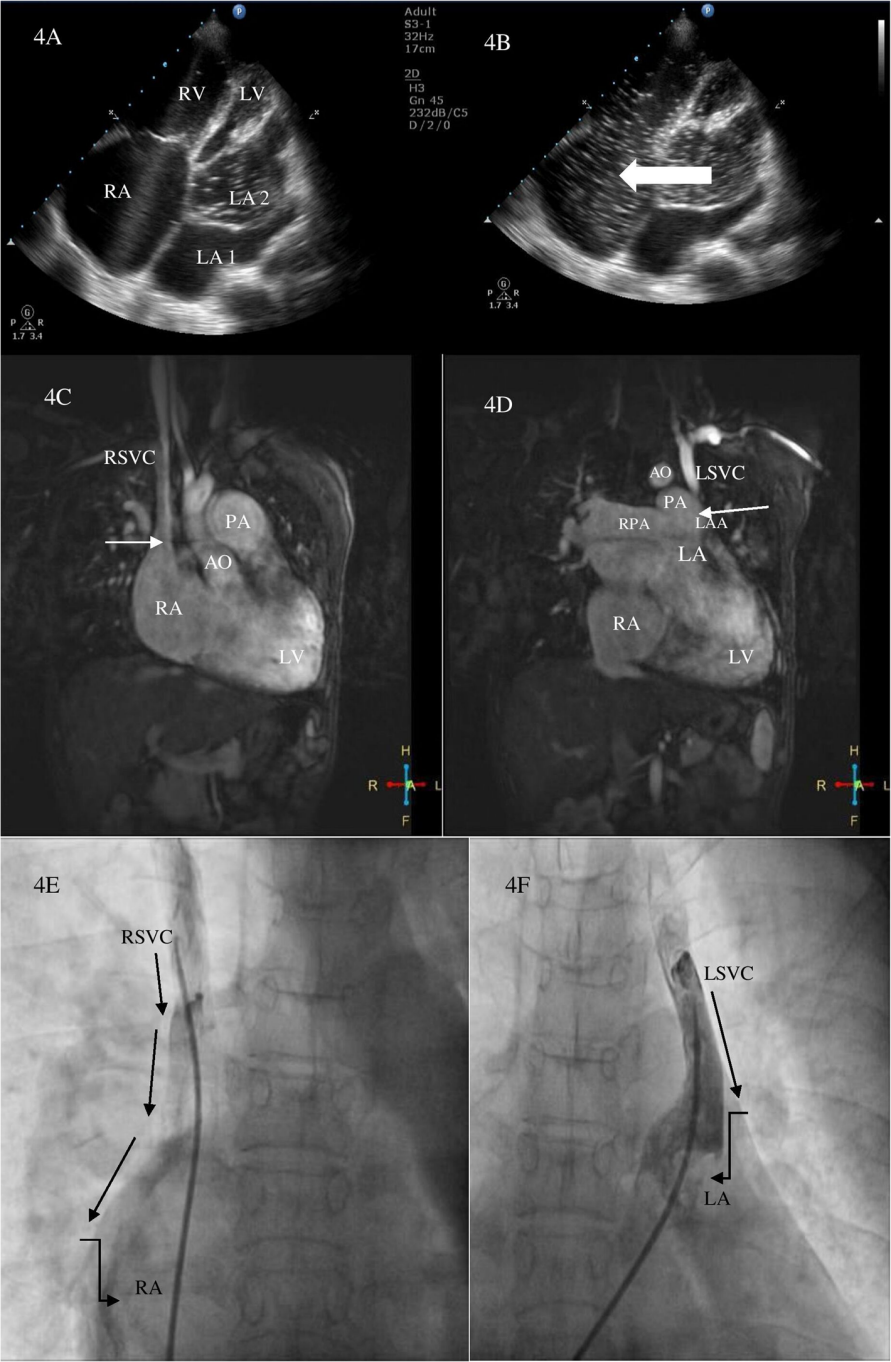
Contrast transthoracic echocardiography in apical 4 chamber view with contrast injected in left antecubital vein showing contrast 1st appearing in LA2 (**A**) and then progressing to right atrium through the ASD (arrow) (**B**). Cardiac MRI showing right superior vena cava draining into right atrium (arrow) (**C**) and left superior vena cava draining into left atrium (arrow) with absence of innominate vein (**D**). Fluoroscopic image with Berman catheter in right superior vena cava showing right superior vena cava draining into right atrium (arrows) (**E**) and left superior vena cava draining into left atrium (arrows) (**F**) confirming completely unroofed coronary sinus. * Indicates completely unroofed coronary sinus. LA, left atrium; RV, right ventricle; LV, left ventricle; RSVC, right superior vena cava; LSVC, left superior vena cava; LAA, left atrial appendage; AO, aorta; PA, pulmonary artery; RPA, right pulmonary artery

**Fig. 5:**
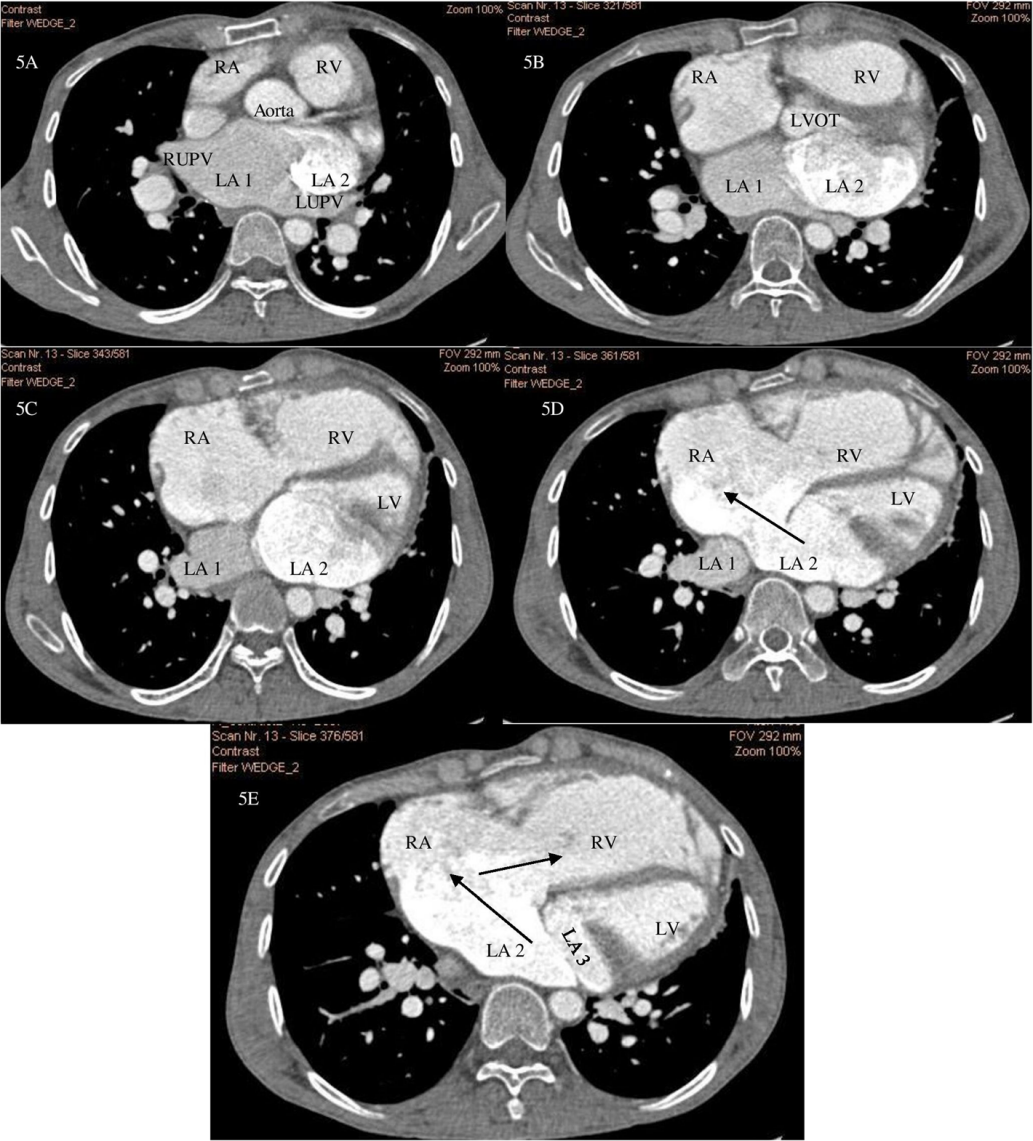
**A**, **B**, **C**, **D**, **E** Cardiac CT with contrast injected in left antecubital vein showing appearance of contrast firstly into middle chamber of LA (LA 2) (**A**) then progressing to RA through ASD (arrows in **D** and **E**) suggestive of PLSVC draining into LA due to completely unroofed coronary sinus confirming presence of Raghib syndrome. Three chambers of LA (labelled LA1, LA 2, and LA3) are clearly visualised. RA, right atrium; LA, left atrium; RV, right ventricle; LV, left ventricle; RUPV, right upper pulmonary vein; LUPV, left upper pulmonary vein; LVOT, left ventricular outflow tract


A 45-year-old gentleman of north Indian ethnicity, teacher by occupation, with no significant medical, family, or psychosocial history presented to us with complaints of bluish discoloration of mucous membranes, breathlessness on exertion belonging to New York Heart Association (NYHA) functional class 3, and palpitations belonging to modified European Heart Rhythm Association (EHRA) class 3 for 1 year. Physical examination revealed central cyanosis and grade three clubbing in all four extremities with oxygen saturation of 82% on room air. Other salient features on physical examination included raised jugular venous pressure with prominent cv waves, grade three parasternal heave, a wide fixed split S2 with loud P2, grade two pansystolic murmur (PSM) at apex, and a grade three PSM at lower left sternal border which increased with inspiration. Twelve lead surface electrocardiogram (ECG) showed right bundle branch block (RBBB) with atrial fibrillation (AF) (Fig. 1A). Chest X-ray showed situs solitus, levocardia, right atrial enlargement, right ventricular (RV) apex, and dilated main and right descending pulmonary arteries with peripheral pruning of pulmonary vessels (Fig. 1B). Two-dimensional transthoracic echocardiography (TTE) revealed dilated right-sided chambers and severe tricuspid regurgitation (TR) with a right ventricular systolic pressure of 100mmHg. TTE also revealed an atrial septal defect (ASD) with maximum size 26.9mm with bidirectional but predominant right to left shunt and two non-obstructive membranes in the LA, dividing the LA into three chambers (Figs. 2 and 3, Videos 1 and 2). The proximal chamber of LA (which we termed LA1) received all four pulmonary veins, the middle chamber (LA2) communicated with the right atrium (RA) through the ASD, and the distal chamber (LA3) communicated with the left ventricle (LV) via the mitral valve (Figs. 2 and 3, Videos 1 and 2). Mitral valve showed presence of accessory tissue, mild mitral regurgitation (MR), and mild mitral stenosis (MS) with a mitral valve area of 1.9cm^2^. The maximum diameter of the proximal membrane was 18.1mm and of the distal membrane was 17.7mm. Transesophageal echocardiography (TEE) confirmed these findings (Video 3). Bubble contrast TTE with contrast injected in left antecubital vein showed contrast first appearing in the middle chamber of LA (LA2), then progressing to RA through the ASD and then to RV, suggesting presence of persistent left superior vena cava (PLSVC) with unroofed coronary sinus (CS) (Fig. 4A, B, Video 4). Contrast cardiac computerized tomography (CT) confirmed these findings (Fig. 5, Video 5). Cardiac magnetic resonance imaging (MRI) showed right superior vena cava (RSVC) draining into RA and PLSVC draining into LA and absence of the innominate vein (Fig. 4 C, D). Cardiac catheterization confirmed these findings (Fig. 4 E, F, Video 6). Catheterization also revealed irreversible severe pulmonary arterial hypertension (PAH) with resistance ratio of 1.1 and step down in the oxygen saturation from pulmonary veins to LA. Through this multimodality imaging approach, we concluded with a diagnosis of cor triatriatum with supramitral ring: “Cor tetratriatum” with Raghib syndrome with Eisenmenger syndrome. Patient was kept on medical management with tablet torsemide 10mg once daily, tablet metoprolol succinate 50mg once daily, tablet warfarin 5mg once daily, and tablet sildenafil 20mg thrice daily. Follow-up at 1 month, 3 months, 6months, and 1 year showed no progression of symptoms, with the patient remaining in NYHA class 2 and modified EHRA class 2A. The international normalised ratio was maintained in the therapeutic range, and he had no adverse effects to any medications.

**Additional file 1: Video 1**. Transthoracic echocardiography in apical 4 chamber view showing left atrium divided into 3chambers by 2 membranes, uninterrupted blood flow from proximal LA chamber to mitral valve, dilated right atrium and right ventricle, presence of tricuspid regurgitation, ASD between middle LA chamber and RA, mild MR with accessory mitral valve tissue over posterior mitral leaflet.


**Additional file 2: Video 2**. Transthoracic echocardiography in parasternal long axis view showing left atrium divided into 3 chambers by 2 membranes, uninterrupted blood flow from proximal LA chamber to mitral valve, dilated right atrium and right ventricle, presence of tricuspid regurgitation, ASD between middle LA chamber and RA, mild MR with accessory mitral valve tissue over posterior mitral leaflet.

## Discussion

Supramitral ring is a type of congenital MS which is supposed to have better prognosis than the other types. It is thought to be derived from failure of the endocardial cushions to divide completely. The ring, which is fibrous, can be complete or partial, maybe annular or supra-annular and maybe associated with normal or abnormal mitral valve [1].

Cor triatriatum sinister is a rare anomaly due to incomplete incorporation of the pulmonary vein into the LA [2]. It results in the formation of two chambers of LA, the posterosuperior chamber which receives blood from pulmonary veins and the anteroinferior chamber which contains the LAA and mitral valve orifice. The size of the opening in the membrane classifies cor triatriatum into three types. Type 1 has no opening, where the accessory left atrium drains into the right heart, type 2 has a small opening, and type 3 has a large opening with little or no obstruction. Types 1 and 2 present in childhood due to obstruction in pulmonary venous return with pulmonary hypertension, while most patients with type 3 are asymptomatic in childhood [2, 3].

Echocardiography is the first-line choice for the evaluation of pulmonary venous anomalies, but it lacks the ability to 3-dimensionally display the pulmonary veins and their relationships to the left atrium. Computed tomography angiography (CTA) is the modality of choice for the diagnosis of pulmonary venous anomalies. It noninvasively and accurately evaluates the presence, course, and number of anomalous veins; associated cardiovascular defects; and any other lung or vascular anomaly with detailed anatomical assessment. It allows rapid acquisition of data with high spatial resolution and wide anatomic coverage. Thus, it is considered superior to echocardiogram and cardiac catheterization. Magnetic resonance imaging (MRI) is an excellent imaging modality for anomalous pulmonary veins. White blood imaging sequences do not always provide sufficient spatial resolution for the more peripheral pulmonary veins, especially when the anatomy is complex. Contrast MRA and time-resolved MRA allow proper visualization of the pulmonary veins; however, it takes a long time. Phase contrast MRI may have a role in evaluation of flow pattern within pulmonary veins. Digital subtraction angiography is a criterion-standard method for assessment of complex congenital heart disease in infants and children, but it is an invasive procedure with high radiation doses [2].

To the best of our knowledge, simultaneous presence of supramitral ring and cor triatriatum has not been described in the literature. Simultaneous presence of both entities in our patient, resulting in formation of four chambers of atria, justifies the term “cor tetratriatum”.

Unroofed coronary sinus is a rare congenital heart defect where the wall of the coronary sinus is partially or completely absent. It is classified according to the Kirklin and Barratt-Boyes classification into four types depending on whether the unroofing is complete or partial, and whether associated with PLSVC or not. The type I defect, completely unroofed CS with PLSVC, is called Raghib syndrome. Though this defect can be made out with TTE/TEE, a better imaging modality is bubble contrast TTE/TEE, where contrast injected in left arm vein results in opacification of LA followed by RA. These findings can be confirmed by cardiac MRI, cardiac CT, and cardiac catheterization [4].

There are few reports on the simultaneous presence of cor triatriatum and Raghib syndrome. It results in the PLSVC draining into lower chamber of LA which communicates with LAA and with RA through the ASD. It predominantly results in right to left shunt, resulting in cyanosis [5].

In our patient, there were two right to left shunts: from PLSVC to LA2 via unroofed coronary sinus and from RA to LA2 through the ASD due to the severe PAH, resulting in cyanosis and clubbing. As both the membranes in the LA were non-obstructive, there was delayed presentation of the patient in the fifth decade of life. Though there was presence of PLSVC with completely unroofed CS and definite right to left shunt from PLSVC to LA2 since birth, presence of associated supramitral ring diverted most of the systemic venous blood entering the middle LA chamber to the RA through the ASD; hence, our patient probably had no history of visible cyanosis before. However, once he developed Eisenmenger syndrome, he developed the second right to left shunt through the ASD resulting in visible cyanosis and clubbing.

## Conclusion

This is the first case report describing the simultaneous presence of supramitral ring and cor triatriatum resulting in a new entity: “cor tetratriatum”. Unlike cor triatriatum with Raghib syndrome, cor tetratriatum with Raghib syndrome presents late with cyanosis as the supramitral ring diverts most of the desaturated blood coming from PLSVC to LA to the RA and presents with cyanosis once Eisenmenger syndrome develops.

## Supplementary Information

**Additional file 3: Video 3.** Transesophageal echocardiography showing left atrium divided into 3 chambers by 2 membranes, presence of ASD with bidirectional shunt, presence of tricuspid and mitral regurgitation.

**Additional file 4:. Video 4.** Contrast transthoracic echocardiography in apical 4 chamber view with contrast injected in left antecubital vein showing contrast first appearing in middle LA chamber and then progressing to right atrium through the ASD and then to right ventricle (arrow).

**Additional file 5: Video 5.** Cardiac CT with contrast injected in left antecubital vein showing appearance of contrast firstly into middle chamber of LA then progressing to RA through ASD (arrow) suggestive of PLSVC drainage to LA due to completely unroofed coronary sinus confirming presence of Raghib syndrome.

**Additional file 6: Video 6.** Fluoroscopic movie with Berman catheter in right superior vena cava showing right superior vena cava draining into right atrium (arrow) and left superior vena cava draining into left atrium (arrow) confirming completely unroofed coronary sinus.

## Data Availability

All data relevant are included in this published article [and its supplementary information files].

## Abbreviations

LAA: Left atrial appendage
NYHA: New York Heart Association
EHRA: European Heart Rhythm Association
PSM: Pansystolic murmur
ECG: Electrocardiogram
RBBB: Right bundle branch block
AF: Atrial fibrillation
RV: Right ventricle
TTE: Transthoracic echocardiography
TR: Tricuspid regurgitation
ASD: Atrial septal defect
LA: Left atrium
RA: Right atrium
MR: Mitral regurgitation
LV: Left ventricle
MS: Mitral stenosis
TEE: Transesophageal echocardiography
PLSVC: Persistent left superior vena cava
CS: Coronary sinus
CT: Computerized tomography
MRI: Magnetic resonance imaging
RSVC: Right superior vena cava
PAH: Pulmonary arterial hypertension
